# Utilizing Triage Data for Medical Imaging Studies in the Emergency Department

**DOI:** 10.7759/cureus.41234

**Published:** 2023-06-30

**Authors:** Maryam H Turkistani, Roaa R Amer

**Affiliations:** 1 Emergency Department, King Abdulaziz Medical City, Riyadh, SAU

**Keywords:** hospital stay, emergency evaluation, radiological image, triage data, emergency department

## Abstract

The use of radiological images is widespread in the emergency department (ED) as physicians commonly rely on them during initial evaluations to confirm diagnoses, contributing to prolonged waiting times. This study aimed to determine the relationship between commonly gathered triage data and the need for radiological imaging. Data were collected from electronic charts that contained routinely collected hospital data at the time of triage in the King Abdulaziz Medical City (KAMC) in Riyadh ED. The binary logistic regression results demonstrated a statistically significant relationship between age and radiological imaging ordered in the ED. Each one-unit increase in age corresponded to a 0.983-fold increase in the likelihood of ordering radiological imaging (odds ratio: 0.983, 95% confidence interval: 0.972-0.995, p = 0.004). In contrast, hypertension, diabetes, and heart failure were independent predictors of the need for radiological imaging in the ED (p >0.05). Patient data that are immediately available during ED triage can be used to predict the need for radiological imaging during ED visits. Such models can identify patients who may require radiological imaging during ED visits and expedite patient disposition.

## Introduction

The utilization of imaging techniques, particularly CT scans, has witnessed a significant rise in the emergency department (ED) in recent years. The primary reason for this surge is the ability of CT scans to effectively confirm or exclude a diagnosis [1-2]. However, the use of these advanced radiological studies is associated with increased ED stay lengths [3-4]. Moreover, ED crowding increases mortality rates in patients [5-6]. Therefore, the development of a physician triage model aimed at improving ED performance by expediting workups while patients are in the waiting area may contribute to decreasing the length of stay and allow ER physicians to see more patients promptly [7]. Such a model can also reduce physician-related factors that were found to play a crucial role in selecting the type of imaging study in the ED [8].

In many cases, radiographic imaging is required for the rapid diagnosis and management of life-threatening diseases. This method is commonly used to establish the disease process and finalize the diagnosis of large groups of patients who present to the ED during their initial evaluations [9-10]. However, there are limited studies regarding the use of immediately available triage data, such as patient age, comorbidities, chief complaints, and number of ER visits, to predict the necessity of emergency imaging studies. The development of such a model can contribute to rapid and informed decisions regarding the need for imaging in the ED [10-11]. Therefore, this study aimed to investigate the relationship between commonly gathered triage data and the need for radiographic imaging in the King Abdulaziz Medical City (KAMC) in Riyadh ED. Discovering such a relationship could expedite the time between patient arrival and imaging studies, ultimately leading to a reduction in the ED length of stay.

## Materials and methods

Study design

This retrospective electronic chart review study used hospital data that had been routinely collected at the time of triage to predict the possibility of admission to the ED.

Study setting and population

This study was approved by the King Abdullah Medical Research Center (KAIMRC) (reference number: RYD-23-419812-70139) and was conducted in the ED of the KAMC in Riyadh. This is the largest ED in the Middle East. The KAMC in Riyadh is a tertiary-care hospital and level I trauma center. The estimated annual number of adult ED visits at the KAMC in Riyadh is 200,000 patients. Ethical consent was not required for this study, as the research was based on data from an electronic medical system.

Study protocol

All adult patients who visited the KAMC in Riyadh ED between January 2018 and December 2019 were included (146,000 patients in total). Patients who were under 14 years old, absconded, triaged away, died on arrival at the ED, or were admitted directly to the general ward or intensive care unit were excluded. Patients with missing data were also excluded.

Patient record numbers were randomly collected using a computer program (the dplyr CRAN package in R, version 1.0.5). Data for each patient were extracted from the Hospital Clinical Information System (BestCare, version 2.0, Korea, ezCaretech), including the triage level determined according to the Canadian Triage and Acuity Scale (CTAS), age, sex, arrival mode, day of presentation, comorbidities, previous ED visits, and previous hospital admissions with the same complaints. All data were collected and coded using Microsoft Excel (Microsoft, Washington, USA). A sample size calculator was used to determine the sample sizes for both datasets at a 5% level (α = 0.01) [12]. The chosen confidence interval (CI) was 95%, the population size was 146,000, and the margin of error was 3%. Furthermore, an ideal sample size of 1,166 was used. The sample size for the derivation dataset was 696 patients (60% of the total sample) while that for the validation dataset was 464 patients (40% of the total sample).

Statistical analysis

All analyses were performed using the SPSS Statistics version 26 (IBM Corp. Released 2019. IBM SPSS Statistics for Windows, Version 26.0. Armonk, NY: IBM Corp.). The normality of continuous variables was assessed using the Kolmogorov-Smirnov test. Continuous variables were reported as mean ± standard deviation, while categorical variables were reported as proportions. The chi-square test was used to compare categorical variables, and the independent sample t-test was used for continuous variables. Binary logistic regression was used to identify the relationship between the need for radiological imaging and the associated risk factors. Odds ratios (OR) and 95% CIs were also determined. Additionally, a decision tree analysis was performed. Statistical significance was defined at p <0.05.

## Results

This study included 1,166 individuals, with a mean age of 46.09 ± 17.04 years. Women constituted a higher proportion (58.60%) than men did (41.40%). Among the participants, 89.60% were Saudi citizens. The previous year’s admissions accounted for 17.4% of the total (Table 1).

**Table 1:** Demographic characteristics of the individuals included in the study

Variables	Scale	Total (n=1166) n (%)
Age (years), mean±SD		46.09±17.04
Gender	Male	483(41.40)
	Female	683(58.60)
Nationality	Saudi	1045(89.60)
	Non-Saudi	61(10.40)
Arrival mode	Ambulance	82(7.00)
	Referral	3(0.30)
	Walk in	1042(89.40)
Triage level/patient acuity	I	5(0.40)
	II	77(6.60)
	III	541(46.40)
	IV	399(34.20)
	V	68(5.80)
Need for radiological imaging		630(54.00)
Admissions in the past 1 year		203(17.40)
Result of visit	Admission	221(19.00)
	Discharge	942(80.78)

Regarding the need for radiological imaging, statistically significant differences were observed between the two groups in terms of age, sex, nationality, total admissions in the previous year, travel mode, triage level, and visit outcomes (p <0.05) (Table 2).

**Table 2:** Comparison between demographic characteristics and the need for radiological imaging in the ED

Variables	Scale	Need for radiological imaging		p-value
		Yes N (%)	No N (%)	
Age (years), mean±SD		47.37±21.55	39.32±16.18	0.001
Gender	Male	280(58.00)	203(42.00)	0.023
	Female	350(51.20)	333(48.80)	
Nationality	Saudi	596(57.00)	449(43.00)	0.001
	Non-Saudi	34(28.10)	87(71.90)	
Admission in the last 1 year	Yes	128(63.10)	75(36.90)	0.010
Arrival mode	Ambulance	74(90.20)	8(9.80)	0.001
	Referral	2(66.70)	1(33.30)	
	Walk in	541(51.90)	501(48.10)	
Triage level	1	3(60.00)	2(40.00)	0.001
	2	71(92.20)	6(7.80)	
	3	335(61.90)	206(38.10)	
	4	165(41.40)	234(58.60)	
	5	15(22.10)	53(77.90)	
Result of visit	Admission	172(77.80)	49(22.20)	0.001
	Discharge	456(48.40)	486(51.60)	

The comparison of comorbidities in relation to the need for radiological imaging revealed a significant difference between the groups that needed radiological imaging and those that did not, particularly with respect to hypertension, diabetes, chronic obstructive pulmonary disease (COPD), and heart failure in the ED. The need for radiological imaging was significantly higher in patients with hypertension and diabetes (36% and 32.5%, respectively, p <0.05), whereas this need was lower in patients with COPD and asthma (1% and 5.4%, respectively) (Table 3).

**Table 3:** Comparison between comorbidities and the need for radiological imaging in the ED COPD, chronic obstructive pulmonary disease; DLP, dyslipidemia

Variables	Scale	Need for radiological imaging		p-value
		Yes N (%)	No N (%)	
Diabetic	Yes	205(32.5)	104(19.4)	0.001
	No	425(67.5)	432(80.6)	
Hypertension	Yes	227(36)	103(19.2)	0.001
	No	403(64)	433(80.8)	
COPD	Yes	6(1.0)	0(0)	0.023
	No	624(99)	536(100)	
Heart failure	Yes	50(7.9)	13 (2.4)	0.001
	No	580(92.1)	523(97.6)	
Asthma	Yes	34(5.4)	34(6.3)	0.492
	No	596(94.6)	502(93.7)	
DLP	Yes	100(15.9)	58(10.8)	0.12
	No	530(94.1)	478(89.2)	

The comparison of chief complaints with the need for radiological imaging in the ED revealed a significant association between the need for radiological imaging and specific symptoms, including chest pain, fever, sore throat, and vaginal bleeding (p <0.05). Among the patients, 88.9% had chest pain, 66.2% had a fever, 17.9% had a sore throat, and 30.8% had vaginal bleeding requiring radiological imaging in the ED (Table 4).

**Table 4:** Comparison between chief complaints and the need for radiological imaging in the ED

Variables	Scale	Need for radiological imaging		p-value
		Yes N (%)	No N (%)	
Chest pain	Yes	64(88.9)	8(11.1)	0.001
	No	566(51.7)	528(48.3)	
Back pain	Yes	11(45.8)	13(54.2)	0.416
	No	619(54.2)	523(45.8)	
Fever	Yes	49(66.2)	25(33.8)	0.030
	No	581(53.2)	511(46.8)	
Epigastric pain	Yes	13(48.1)	14 (51.9)	0.535
	No	617(54.2)	522(45.8)	
Sore throat	Yes	7(17.9)	32(82.1)	0.001
	No	623(55.3)	504(44.7)	
Vaginal bleeding	Yes	8(30.8)	18(69.2)	0.016
	No	622(54.6)	518(45.4)	
Palpitation	Yes	7(41.2)	10(58.8)	0.284
	No	623(54.2)	526(45.8)	

The binary logistic regression results demonstrated a statistically significant relationship between age and radiological imaging ordered in the ED. For every one-unit increase in age (OR: 0.983, 95% CI: 0.972-0.995, p = 0.004), there was a 0.983-fold increase in the likelihood of radiological imaging being ordered in the ED. In contrast, hypertension, diabetes, and heart failure were independent predictors of the need for radiological imaging in the ED (p >0.05) (Table 5).

**Table 5:** Predictor variables for the need for radiological imaging based on the binary logistic regression results OR, odds ratio; CI, confidence interval

Variable		OR	(95% CI)	p-value
Age		0.983	(0.972-0.995)	0.004
Hypertension	Yes	1.286	(0.755-2.192)	0.354
Diabetes	Yes	1.116	(0.684-1.820)	0.661
Heart failure	Yes	0.534	(0.273- 1.044)	0.182

**Figure 1:**
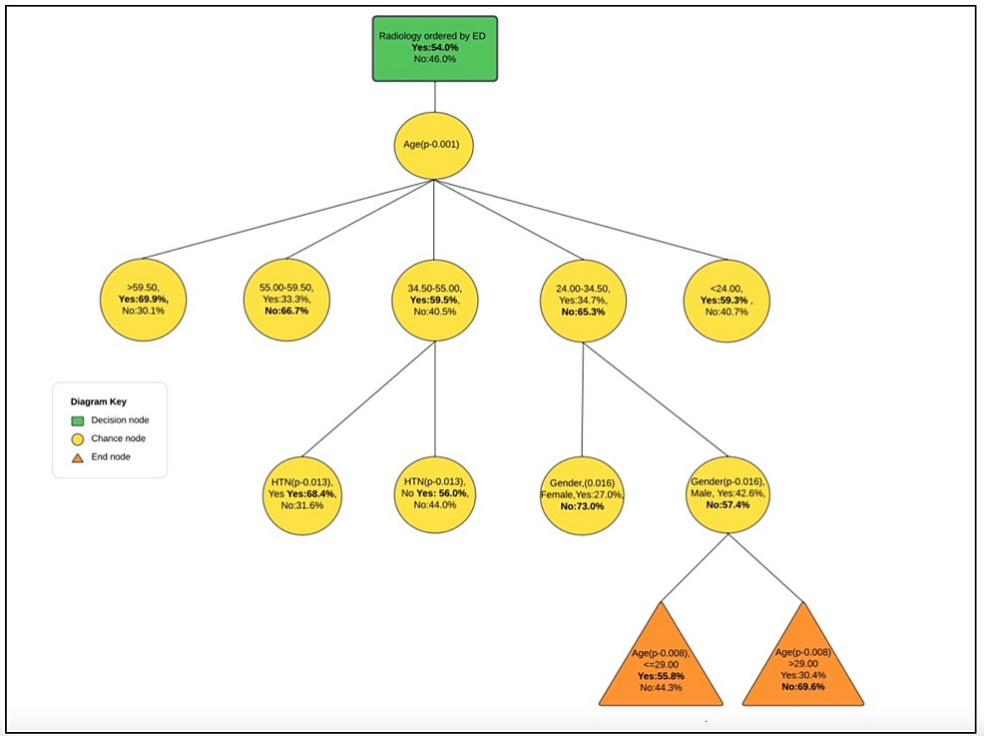
Decision tree analysis diagram

According to the decision tree analysis, age (p = 0.001, χ² = 84) was the most significant factor in determining the need for radiological imaging in the ED. As we examine the branches of the tree, we observe that hypertension became the next crucial factor for individuals aged 35.50-55.00 years who required radiological imaging (p = 0.013, χ² = 6), and sex was important for those aged 24.00-34.50 years. Notably, age was the most significant factor for men (Figure 1). The classification table summarizes the percentages of correct classification, indicating that the model correctly classified 85.90% of the individuals who required radiological imaging. However, it also indicates that 38.10% of those who did not require imaging were incorrectly classified. Overall, our predictions were accurate for 63.90% of the cases (Table 6).

**Table 6:** Classification results of the decision tree analysis Observed; underwent radiological imaging with hypertension and individuals aged 35.50-55.00

Observed	Predicted		
	Yes	No	Percent correct
Yes	541	89	85.90%
No	332	204	38.10%
Overall percentage	74.90%	25.10%	63.90%

## Discussion

In this study, data commonly provided by patients in the triage phase was used to predict the need for radiological imaging. By developing a decision tree diagram, we identified age as the most significant factor in determining the need for radiological imaging in the ED, followed by hypertension for those aged 35.50-55.00 years (p = 0.013, χ² = 6) and sex for those aged 24.00-34.50 years. Notably, age was the most significant factor for men. The decision tree, developed using administrative triage data collected during the initial interaction with the ED, proved to be a valuable tool for predicting outcomes. This predictive model has the potential to contribute to the reduction of length of stay and alleviate ED crowding. By assisting physicians in making rapid decisions regarding the need for radiological imaging, the model can expedite the diagnostic process and facilitate timely interventions.

Numerous efforts have been made to reduce the utilization of imaging in the ED. The National Council on Radiation Protection and Measurements [12] and the American College of Radiology have developed guidelines to help physicians in deciding the best radiological modality for each specific clinical case, thereby improving the quality of care [13]. However, these guidelines primarily depend on physician assessments. In our study, we aimed to expedite the process of predicting radiological needs using administrative triage data. Our findings align with other studies, indicating that as age increases, the likelihood of requiring radiological imaging also increases. Furthermore, our study revealed a higher likelihood of imaging needs among patients triaged as CTAS 1 and 2, compared to that in patients triaged as CTAS 3-5 [14-15].

This study had some limitations. First, this study was confined to a single center in a capital city for one year, which can make generalizing the results to other centers difficult. It is important to note that different hospitals may not have similar administrative triage data or electronic record systems during the triage phase. Second, the sample size used for developing the decision tree was limited due to the exclusion of data from absconded patients. This small sample size may impact the reliability and generalizability of the results. Third, the study did not investigate the specific reasons for patients needing imaging or differentiate between different types of radiological imaging. Fourth, as a retrospective study, there is a possibility of selection bias and unmeasured confounding factors that were not accounted for. Lastly, the study was unable to determine whether patients were offered radiological imaging and whether they accepted or refused.

## Conclusions

In this study, we developed a decision tree diagram to help predict the eventual use of radiological imaging using only the information gathered during ED triaging, including patients’ demographic data, general medical information, and chief complaints. We found out that age is the most significant factor followed by hypertension. This decision tree can aid in predicting the need for radiological imaging, allowing for timely referral to the radiology department and potentially reducing ED waiting times.
